# The KCa3.1 blocker TRAM34 reverses renal damage in a mouse model of established diabetic nephropathy

**DOI:** 10.1371/journal.pone.0192800

**Published:** 2018-02-09

**Authors:** Chunling Huang, Ling Zhang, Ying Shi, Hao Yi, Yongli Zhao, Jason Chen, Carol A. Pollock, Xin-Ming Chen

**Affiliations:** 1 Kolling Institute, Sydney Medical School-Northern, University of Sydney, Royal North Shore Hospital, St Leonards, New South Wales, Australia; 2 School of Pharmaceutical Science &Yunnan Key Laboratory of Pharmacology for Natural Products, Kunming Medical University, Kuming, China; 3 Department of Pediatrics, the Second Hospital of Dalian Medical University, Dalian, Liaoning, China; 4 Department of Anatomical Pathology, Royal North Shore Hospital, St Leonards, Sydney, New South Wales, Australia; Max Delbruck Centrum fur Molekulare Medizin Berlin Buch, GERMANY

## Abstract

Despite optimal control of hyperglycaemia, hypertension, and dyslipidaemia, the number of patients with diabetic nephropathy (DN) continues to grow. Strategies to target various signaling pathways to prevent DN have been intensively investigated in animal models and many have been proved to be promising. However, targeting these pathways once kidney disease is established, remain unsatisfactory. The clinical scenario is that patients with diabetes mellitus often present with established kidney damage and need effective treatments to repair and reverse the kidney damage. In this studies, eNOS-/- mice were administered with streptozotocin to induce diabetes. At 24 weeks, at which time we have previously demonstrated albuminuria and pathological changes of diabetic nephropathy, mice were randomised to receive TRAM34 subcutaneously, a highly selective inhibitor of potassium channel KCa3.1 or DMSO (vehicle) for a further 14 weeks. Albuminuria was assessed, inflammatory markers (CD68, F4/80) and extracellular matrix deposition (type I collagen and fibronectin) in the kidneys were examined. The results clearly demonstrate that TRAM34 reduced albuminuria, decreased inflammatory markers and reversed extracellular matrix deposition in kidneys via inhibition of the TGF-β1 signaling pathway. These results indicate that KCa3.1 blockade effectively reverses established diabetic nephropathy in this rodent model and provides a basis for progressing to human studies.

## Introduction

Diabetic nephropathy is the leading cause of end-stage renal failure, accounting for 35–40% of all new cases requiring dialysis therapy throughout the world. The World Health Organization estimates that globally 415 million adults have diabetes, which will rise to 642 million by 2040 [1]. The increasing incidence of diabetes elevates diabetic nephropathy to one of the most important current public health issues, representing a significant burden on the health system. Currently, the clinical management of diabetic kidney disease includes optimal blood pressure and glycaemic control, and therapies to target a reduction in albuminuria [2]. However, such strategies have only slowed the progression to end-stage kidney disease (ESKD). Preventing the onset or development of diabetic nephropathy through targeting various signaling pathways of inflammation and fibrosis have been intensively investigated in animal models and many are effective [3]. However, strategies to reverse established diabetic nephropathy, which are more relevant to the clinical situation, have not been established.

It is well accepted that chronic hyperglycaemia activates various inflammatory pathways to induce oxidative stress, fibrotic cytokines including transforming growth factor β-1 (TGF-β1), the renin-angiotensin-aldosterone system, and increases advanced glycation end-products, leading collectively to tubular and podocyte injury, apoptosis, extracellular matrix deposition and associated albuminuria [4, 5]. Inflammation plays a critical role in the pathogenesis of diabetic nephropathy and inflammation related molecules and pathways including cell adhesion molecules, growth factors, chemokines and pro-inflammatory cytokines are critically involved in the progression of diabetic nephropathy [6]. The replacement of renal architecture by extracellular matrix correlates closely with the progressive loss of renal function [7, 8].

The calcium-activated potassium channel KCa3.1 is part of a potential heterotetrameric voltage-independent potassium channel which is activated by intracellular calcium. The activation is followed by membrane hyperpolarization, which promotes calcium influx. KCa3.1 regulates membrane potential and calcium signalling in various types of cells. KCa3.1-mediated Ca2+ influx is associated with inflammation, atherogenesis, and proliferation of endothelial cells, T lymphocytes, macrophages, and fibroblasts [9]. It has been reported that blocking KCa3.1 suppresses plaque instability in advanced stages of atherosclerosis by inhibiting macrophage polarization [10]. KCa3.1 has been suggested as a potential therapeutic target for diseases including kidney fibrosis [11], ulcerative colitis [12], hypertension, restenosis and atherosclerosis [13], asthma [14], cancer, autoimmune disorders and vascular inflammation [15]. The KCa3.1 selective blocker Senicapoc has been used to treat patients with sickle cell disease in a clinical trial. In this randomized, double-blind, placebo-controlled phase I trial in healthy volunteers, Senicapoc effectively blocked KCa3.1 channels without significant adverse effects [16]. Subsequently a 12-week, multi-center, randomized double-blind Phase II clinical trial showed that Senicapoc reduced hemolysis and increased hemoglobin levels [17]. Senicapoc may exert its effects through inhibiting the calcium dependent flux of potassium [18]. Renal fibrosis induced by unilateral ureteral obstruction in mice is paralleled by robust upregulation of Kca3.1 in affected kidneys, and selective pharmacologic blockade of Kca3.1 attenuated progression of obstruction-induced renal fibrosis [19]. We have previously shown that blockade of KCa3.1 prevented the development of extracellular matrix deposition and fibrosis in diabetic nephropathy through inhibition of the TGF-β1/Smad signaling pathway [11], limiting activation of renal fibroblasts [20], suppression of TGF-β1 induced monocyte chemoattractant protein-1 (MCP-1) expression and high glucose induced chemokine (C-C motif) ligand 20 (CCL20) expression in renal proximal tubular cells [21, 22]. In addition, we also demonstrated that KCa3.1 mediated dysfunction of tubular autophagy in diabetic kidneys via the PI3k/Akt/mTOR signaling pathways [23].

The current study has examined whether blockade of KCa3.1 is able to reverse established renal damage caused by diabetes in the eNOS-/- mouse model. The results have demonstrated that pharmacological inhibition of KCa3.1 by TRAM34 restored impaired renal injury and significantly diminished inflammatory and fibrotic responses in kidneys from mice with established diabetic nephropathy.

## Materials and methods

### Materials

The selective KCa3.1 blocker TRAM34 (1-[(2-chlorophenyl) diphenylmethyl]-1H-pyrazole) was purchased from Sigma-Aldrich (St. Louis, MO). KCa3.1 antibody was purchased from Abnova (Taiwan). CD68 and F4/80 antibodies were obtained from AbD Serotec (Oxford, UK). Type I collagen antibody was obtained from Abcam (Cambridge, MA). Fibronectin antibody was from Sigma (St. Louis, MO). TGF-β1 and phospho-Smad2/3 antibodies were purchased from LifeSpan (Seattle, WA) and Santa Cruz Biotechnology, CA.

### Methods

#### Animal studies

Eight-week-old male eNOS-/-mice (Jackson laboratory, ME) weighing approximately 20 to 25g were assigned to receive either 5 doses of 55 mg/kg of streptozotocin (STZ) (Sigma, MO) diluted in 0.1 M citrate buffer, pH 4.5, or citrate buffer alone by intraperitoneal injection as described previously [11]. The eNOS-/- mice receiving citrate buffer alone served as non-diabetic controls. At 24 weeks of diabetes, eNOS-/- mice with diabetic nephropathy were then randomized into two groups, to receive TRAM34 (Sigma), 120 mg/kg/day subcutaneously or vehicle (DMSO) for 14 weeks. All animals were housed in the Kearns Animal Facility of Kolling Institute with a stable environment maintained at 22 ± 1°C with a 12/12-h light-dark cycle.

Mice were weighed and their blood glucose levels were measured using Accu-chek glucometer (Roche Diagnostics) weekly and only STZ-treated animals with blood glucose >16 mmol/l were considered diabetic. Diabetic mice received insulin (Lantus, Germany) treatment to prevent ketosis. Mice were euthanized by cervical dislocation under anesthesia with 2% isoflurane, nitrous oxide (2L/min) and oxygen (1L/min). At the time of sacrifice, 24-hour urine was collected and body weight recorded. Urine albumin and creatinine levels were determined with Murine Microalbuminuria ELISA kit and Creatinine Companion kit (Exocell Inc., Philadelphia, PA). After animals were euthanized, left kidneys were removed and snap frozen for the isolation of RNA, and right kidneys were perfused with PBS and fixed in 10% buffered formalin for histological examination.

This study was carried out in strict accordance with the recommendations in the Guide of the National Health and Medical Research Council of Australia's Code for the Care and Use of Animals for Scientific Purposes. The protocol was approved by the Animal Research Ethics Committee of Royal North Shore Hospital.

#### Histology and immunohistochemistry

Paraffin-embedded kidney sections were used for immunohistochemical staining. Matrix deposition within the interstitium was assessed using Masson’s trichrome stain (American MasterTech, Lodi, CA). Briefly, endogenous peroxidase activity was blocked by incubation in 0.3% hydrogen peroxide. After pre-incubation with 10% protein block (Dako, CA) for 10 minutes at room temperature to block nonspecific binding of antibodies, the tissues were incubated overnight at 4°C with primary antibodies against KCa3.1, CD68, F4/80, type I collagen, fibronectin, TGF-β1 and p-Smad2/3. After incubation with appropriate secondary antibodies, sections were developed with 3, 3-diaminobenzidine (Dako, CA) to produce a brown colour and counterstained with haematoxylin. Positive signals in the renal cortex regions were quantified using Image J software as previously described [11]. The number of cells positive for CD68+, F4/80+, phospho-Smad2/3+ were counted in 10 high power fields (HPF, 40×) of the tubulointerstitium.

#### RNA isolation and RT-PCR analysis

Total RNA was extracted from mouse kidneys using Trizol (Invitrogen, CA) respectively. The cDNA was synthesized using the Transcriptor First Strand cDNA Synthesis Kit (Roche). Quantitative real-time PCR was performed using the SYBR Green PCR master mix kit (Roche) with the intron-spanning primers on ABI-Prism-7900 Sequence Detection System (Applied Biosystems). The PCR primer sets for mice β-actin: (forward) 5’-TGACAGGATGCAGAAGGAGA-3’, (reverse) 5’-CGCTCAGGAGGAGCAATG-3’. Type I collagen: (forward) 5’-CATGTTCAGCTTTGTGGACCT-3’, (reverse) 5’- GCAGCTGACTTC AGGGATGT-3’. Fibronectin: (forward) 5’-CGGAGAGAGTGCCCCTA CTA-3’, (reverse) 5’-CGATATTGGTGAATCGCAGA-3’, TGF-β1: (forward) 5’-TCAGA CATTCGGGAAGCAGT-3’, (reverse) 5’- ACGCCAGGAATTGTTGCTAT-3’. The reaction conditions consisted of 1 cycle of initial denaturation at 95°C for 5 min, followed by 45 cycles of 95°C for 15 seconds and 60°C for 1 min. Reaction specificity was confirmed by melting curve analysis. The relative mRNA expression levels were calculated according to the 2^_ΔΔCt^ method. The mRNA expression of β-actin was used as endogenous reference control.

#### Statistical analysis

Statistical analysis of data from multiple groups was compared by one-way ANOVA, followed by Tukey post test (GraphPad Prism 7 software, La Jolla California USA). Statistical significance was determined as *P* < 0.05.

## Results

### KCa3.1 blocker TRAM34 reduced upregulated KCa3.1 expression in mice with established diabetic nephropathy

**Fig 1:**
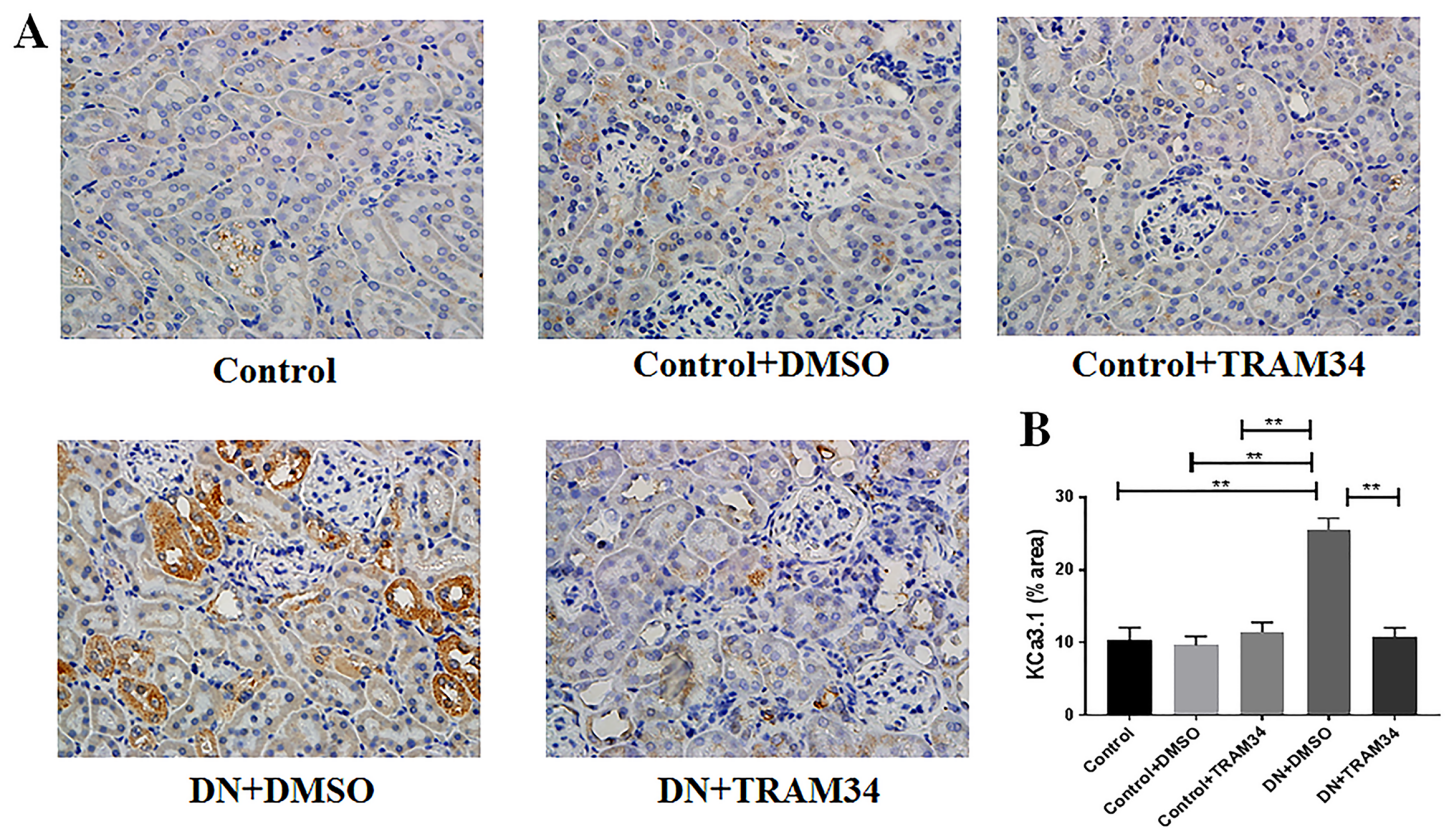
KCa3.1 blocker TRAM34 reduced upregulated KCa3.1 expression in mice with established diabetic nephropathy. **A**. Immunohistochemical analysis showed increased KCa3.1 expression in vehicle-treated diabetic nephropathy mice compared to control mice, which was reduced in diabetic nephropathy mice treated with TRAM34. **B**. The quantitation of KCa3.1 expression in mice kidneys. Results are presented as mean ± SEM. **P<0.01. Original magnification: × 400. N = 5–8.

To investigate whether KCa3.1 is involved in established diabetic nephropathy, the expression of KCa3.1 in eNOS-/- mice with established diabetic nephropathy was examined using immunohistochemical analysis. The diabetic eNOS-/- mouse model is endorsed by the Animal Models of Diabetic Complications Consortium as an appropriate model of diabetic nephropathy [24]. As shown in Fig 1A, a low basal level of KCa3.1 expression was observed in kidneys of control mice (Control), which was significantly upregulated in vehicle-treated diabetic nephropathy mice (DN+DMSO) (*P*<0.01). The administration of TRAM34 significantly reversed the upregulated KCa3.1 expression in diabetic nephropathy (DN+TRAM34) compared to that of vehicle-treated mice (DN+DMSO) (*P*<0.01, Fig 1B). These data indicate a potential pathophysiological role of the KCa3.1 channel in established diabetic nephropathy.

### KCa3.1 blocker TRAM34 ameliorated the renal injury in mice with established diabetic nephropathy

To examine the therapeutic effect of KCa3.1 in established diabetic nephropathy *in vivo*, the eNOS-/- mice with established diabetic nephropathy were treated with or without TRAM34. As shown in Table 1, mice with diabetic nephropathy showed significantly increased blood glucose levels and reduced body weight compared with control group (*P*<0.01). TRAM34 did not affect blood glucose levels or weight gain.

**Table 1:** Metabolic and physiological parameters of eNOS-/- control and diabetic nephropathy mice treated with vehicle (DMSO) and TRAM34.

	Control	Control +DMSO	Control +TRAM34	DN +DMSO	DN +TRAM34
**Blood Glucose Level (mmol/L)**	10.46±0.237	10.69±0.366	11.02±0.325	25.96 ± 0.467*	25.81 ± 0.531*
**Body Weight (g)**	31.32±0.765	31.28±0.816	31.17±0.876	19.1±0.735*	21.04±0.447*
**24h Albumin (mg/24h)**	15.68±3.461	21.23±2.565	13.77± 6.347	49.19± 11.01*	23.89± 4.31^#^
**Albumin/Creatinine Ratio (mg/mg/24h)**	0.11+0.03	0.18+0.03	0.18+0.08	4.25+1.80*	2.26+1.02

Data are presented as mean ± SEM.

* *P*<0.01, vs Control,

^#^*P*<0.05, vs DN+DMSO.N = 5–8.

To determine renal injury, 24h urine albumin excretion and albumin/creatinine ratio was measured at the time of sacrifice. Urine albumin excretion was 15.68±3.461 mg/24 hours in the control group (Control) and increased to 49.19±11.01 mg/24 hours (*P*< 0.01) in mice with diabetic nephropathy (DN+DMSO). This effect was significantly reduced in diabetic nephropathy mice treated with TRAM34, to 23.89±4.31 mg/24 hours (DN+TRAM34) (*P*< 0.05, versus DN+DMSO). Similar results were found in albumin/creatinine ratio. These results indicate that the KCa3.1 blocker TRAM34 ameliorated renal injury in the mice with established diabetic nephropathy, implicating a causative role of KCa3.1 in STZ-induced diabetic nephropathy.

### KCa3.1 blocker TRAM34 prevented macrophage infiltration in mice with established diabetic nephropathy

**Fig 2:**
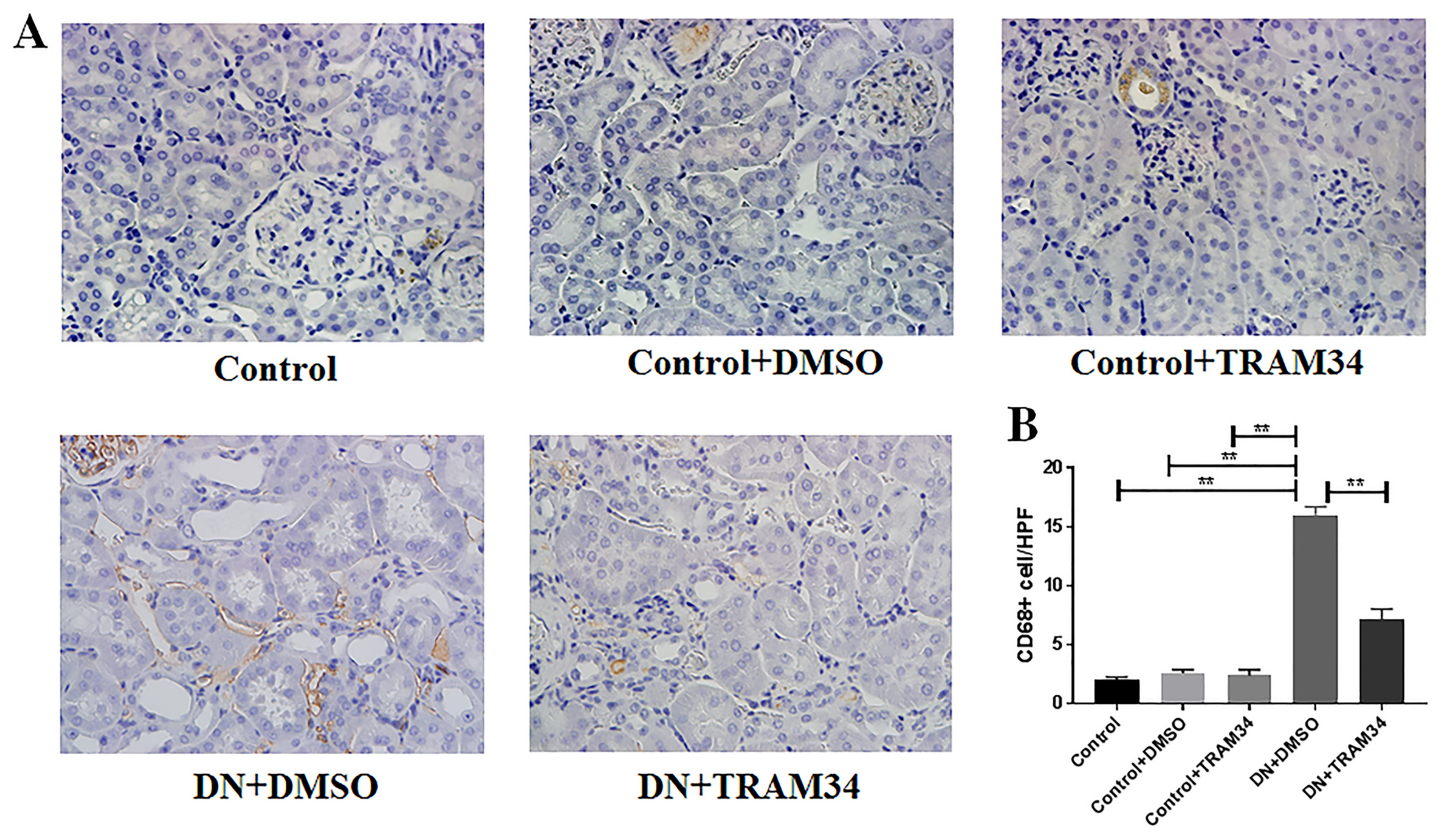
KCa3.1 blocker TRAM34 reduced increased CD68+ cells in mice with established diabetic nephropathy. A. Immunohistochemical analysis showed an increased number of CD68+ cells in mice with diabetic nephropathy compared to control mice, which was reduced in diabetic nephropathy mice treated with TRAM34. B. The quantitation of CD68+ cells in mice kidneys. Results are presented as mean ± SEM. *P<0.05 and **P<0.01. Original magnification: × 400. N = 5–8.

**Fig 3:**
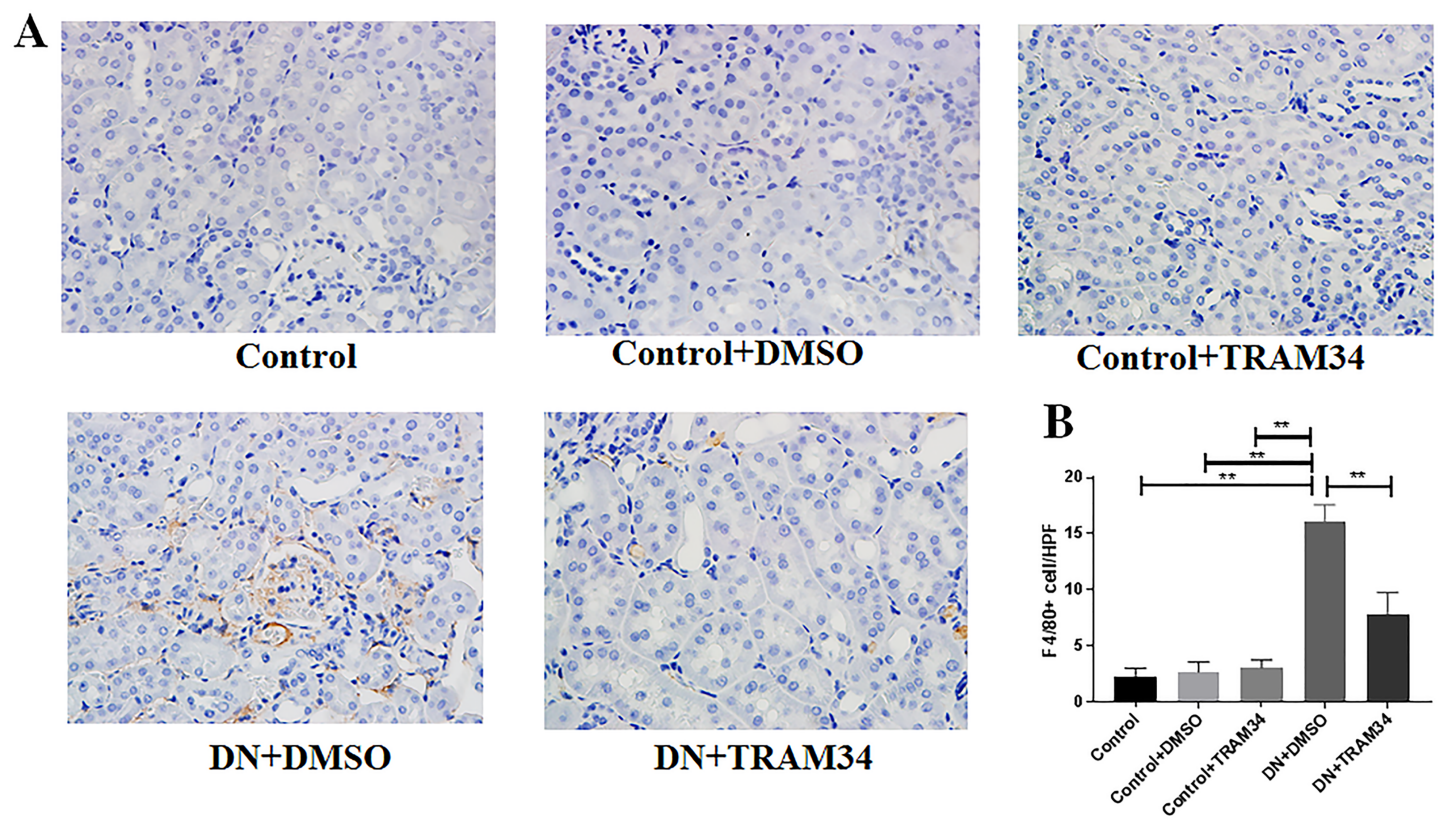
KCa3.1 blocker TRAM34 reduced increased F4/80+ cells in mice with established diabetic nephropathy. A. Immunohistochemical analysis showed increased number of F4/80+ cells in vehicle-treated diabetic nephropathy mice compared to control mice, which was reduced in mice with diabetic nephropathy treated with TRAM34. B. The quantitation of F4/80+ cells in mice kidneys. Results are presented as mean ± SEM. *P<0.05 and **P<0.01. Original magnification: × 400. N = 5–8.

To characterize the role of KCa3.1 in the regulation of inflammation in established diabetic nephropathy, two macrophage markers CD68 and F4/80 were examined in mice kidney tissues using immunohistochemical analysis. The number of CD68+ cells were significantly increased in the mice with diabetic nephropathy (DN+DMSO) when compared with controls (Control) (Fig 2, *P*<0.01). Treatment with TRAM34 (DN+TRAM34) significantly reduced the increased CD68+ cells in mice with diabetic nephropathy (Fig 2, *P*<0.01). Consistent with this finding, we also observed a significant reduction of F4/80+ cells in mice with diabetic nephropathy treated with TRAM34 (DN+TRAM34) (*P*<0.01, Fig 3). These data suggest that KCa3.1 contributed to macrophage infiltration in mice with established diabetic nephropathy.

### KCa3.1 blocker TRAM34 reduced extracellular matrix deposition in mice with established diabetic nephropathy

**Fig 4:**
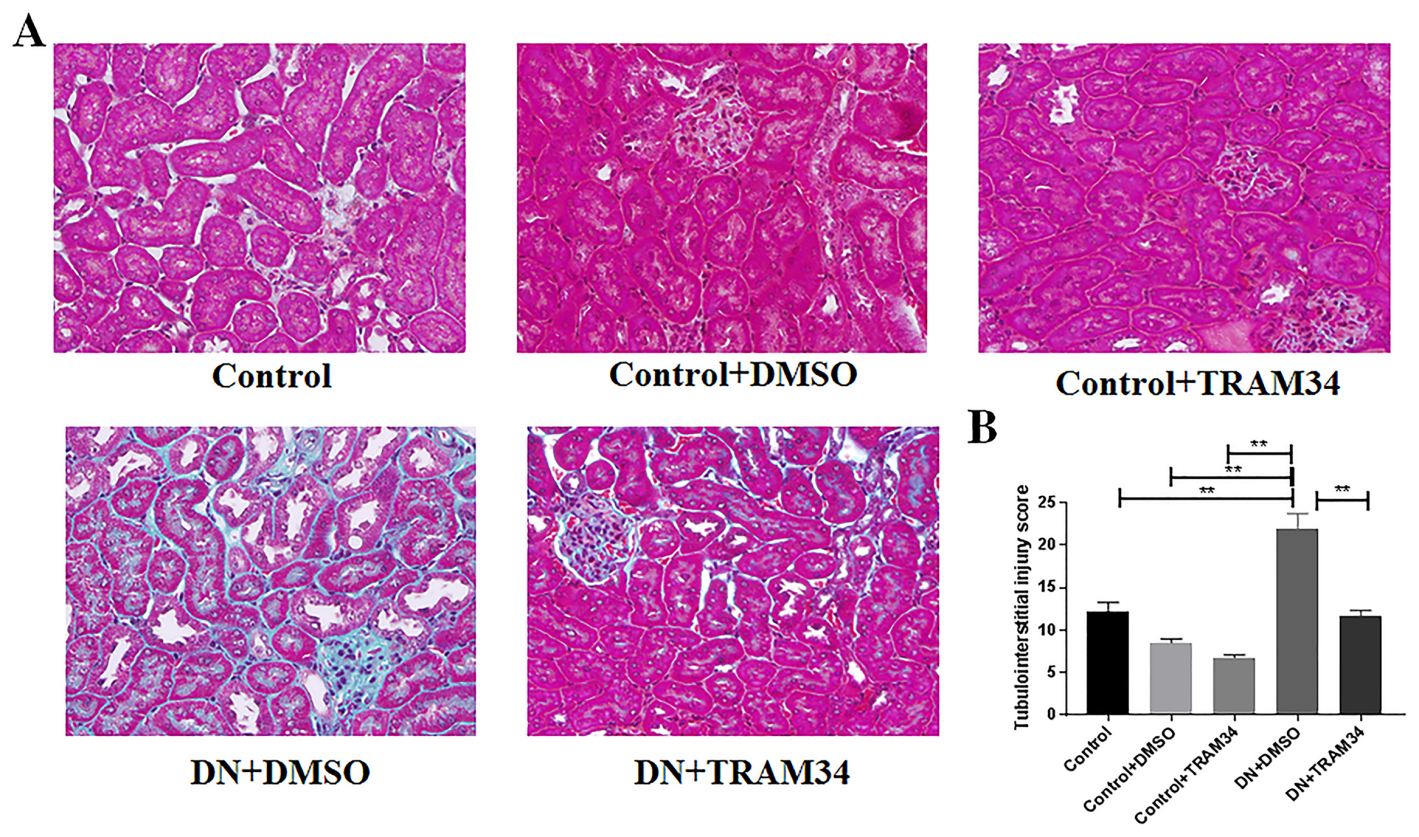
KCa3.1 blocker TRAM34 suppressed the collagen deposition in mice with established diabetic nephropathy. **A**. Masson's trichrome staining showed an increase in collagen accumulation and deposition within the tubulointerstitium in vehicle-treated diabetic nephropathy mice compared to control mice, which was suppressed in diabetic nephropathy mice treated with TRAM34. **B**. The the tubulointerstitial injury score in mice kidneys. Results are presented as mean ± SEM. **P<0.01. Original magnification: × 400. N = 5–8.

**Fig 5:**
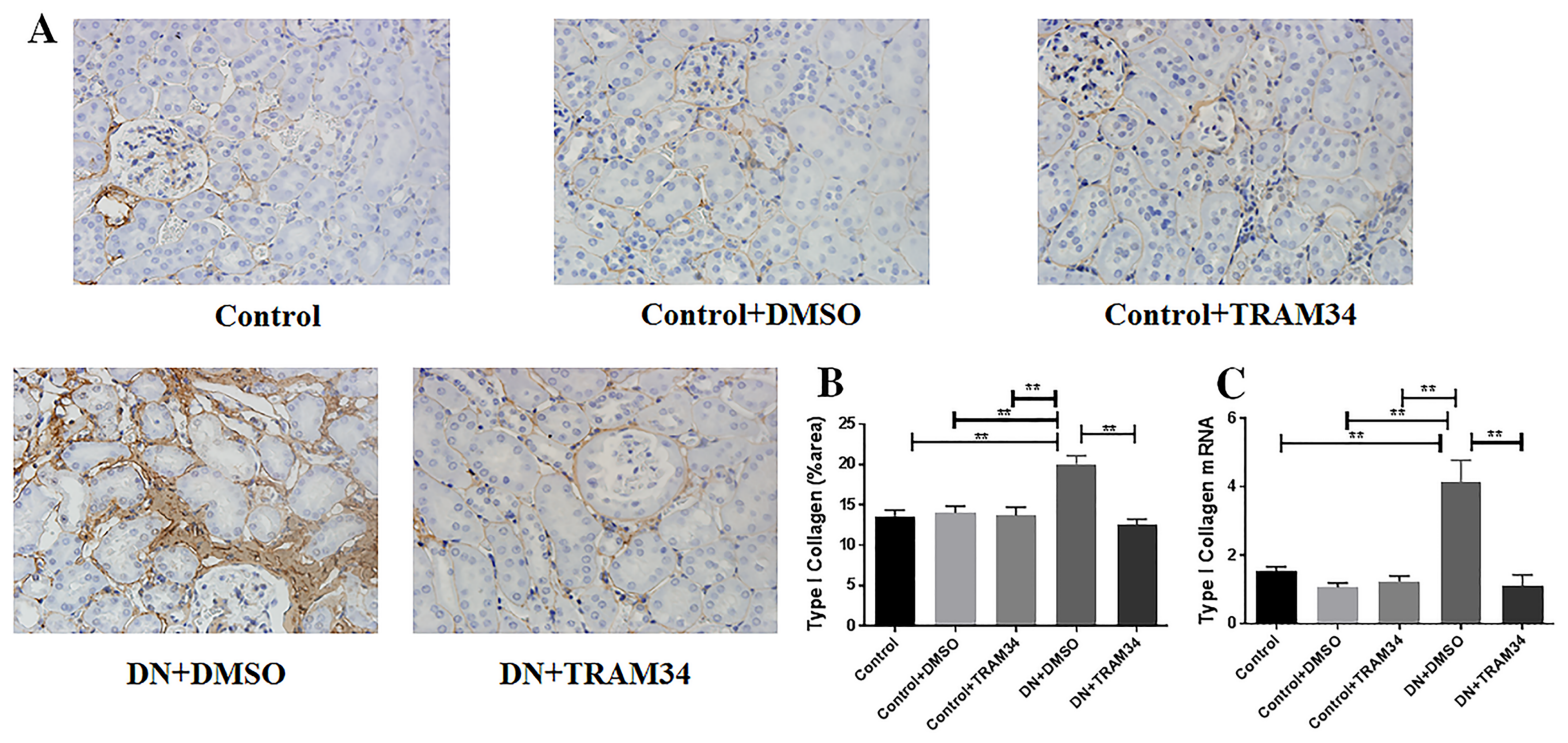
KCa3.1 blocker TRAM34 suppressed the upregulation of type I collagen in mice with established diabetic nephropathy. **A**. Immunohistochemical analysis showed increased type I collagen expression in mice with diabetic nephropathy mice compared to control mice, which was suppressed when mice were treated with TRAM34. **B**. The quantitation of type I collagen expression in mice kidneys. **C**. Quantitative RT-PCR showed increased mRNA expression of type I collagen in mice with diabetic nephropathy compared to control mice but reduced with TRAM34 treatment. Results are presented as mean ± SEM. **P<0.01. Original magnification: × 400. N = 5–8.

**Fig 6:**
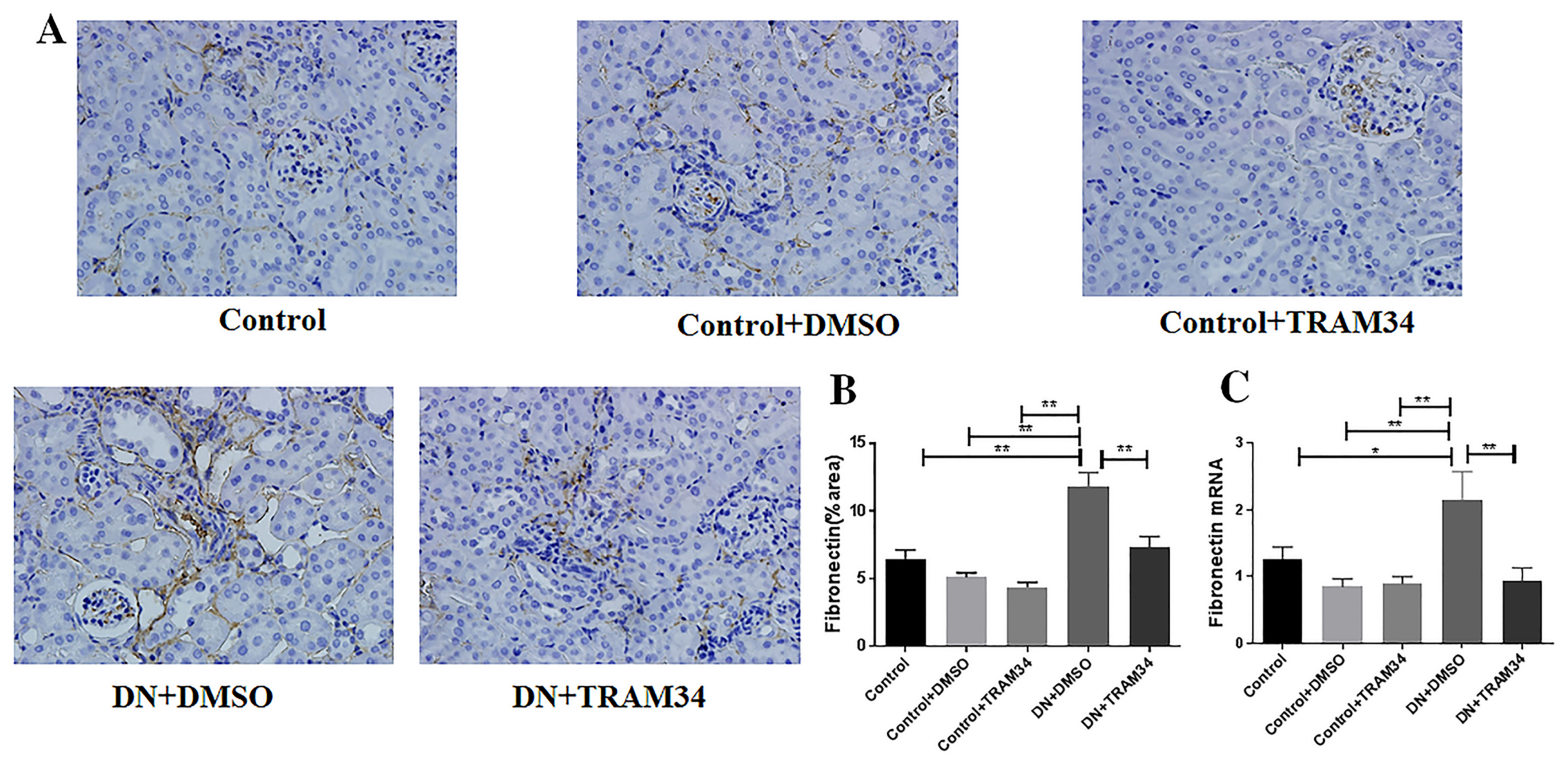
KCa3.1 blocker TRAM34 suppressed the upregulation of fibronectin in mice with established diabetic nephropathy. **A**. Immunohistochemical analysis showed increased fibronectin expression in mice with diabetic nephropathy compared to control mice, which was suppressed when treated with TRAM34. **B**. The quantitation of fibronectin expression in mice kidneys. **C**. Quantitative RT-PCR showed increased mRNA expression of fibronectin in mice with diabetic nephropathy compared to control mice but reduced with TRAM34 treatment. Results are presented as mean ± SEM. *P<0.05, **P<0.01. Original magnification: × 400. N = 5–8.

To determine whether KCa3.1 is involved in the regulation of renal fibrosis in established diabetic nephropathy, we evaluated the effect of KCa3.1 on the expression of interstitial collagen fibrils by Masson's trichrome staining, type I collagen and fibronectin by immunohistochemical staining. As indicated in Fig 4, an increase in collagen accumulation and deposition was observed within the tubulointerstitium after induction of diabetes in vehicle-treated diabetic nephropathy mice (DN+DMSO). The administration of TRAM34 significantly reversed tubulointerstitial damage in diabetic kidneys as compared with vehicle-treated diabetic nephropathy mice (DN+DMSO) (*P* <0.01). Furthermore, the immunohistochemical staining showed substantially increased expression of type I collagen (*P*<0.01, Fig 5) and fibronectin (*P*<0.01, Fig 6) in the vehciel-treated diabetic nephropathy mice (DN+DMSO) when compared with controls (Control), which were significantly reversed after TRAM34 treatment. In addition, we also examined the effect of KCa3.1 on the expression of type I collagen and fibronectin by real time PCR. In line with those observations, gene expression levels of fibrotic markers (type I collagen and fibronectin) were considerably increased in vehcile treated diabetic nephropathy mice (DN+DMSO) when compared to controls (Control) (*P*<0.05, Figs 5C and 6C). The treatment of TRAM34 (DN+TRAM34) significantly attenuated this response in diabetic nephropathy mice as compared with the vehicle-treated diabetic nephropathy mice (DN+DMSO) (*P*<0.01, Figs 5C and 6C). These results indicate that blockade of KCa3.1 with TRAM34 suppressed extracellular matrix deposition in established diabetic nephropathy.

### KCa3.1 blocker TRAM34 inhibited the expression of TGF-β1, and phosphorylation of Smad2/3 in mice with established diabetic nephropathy

**Fig 7:**
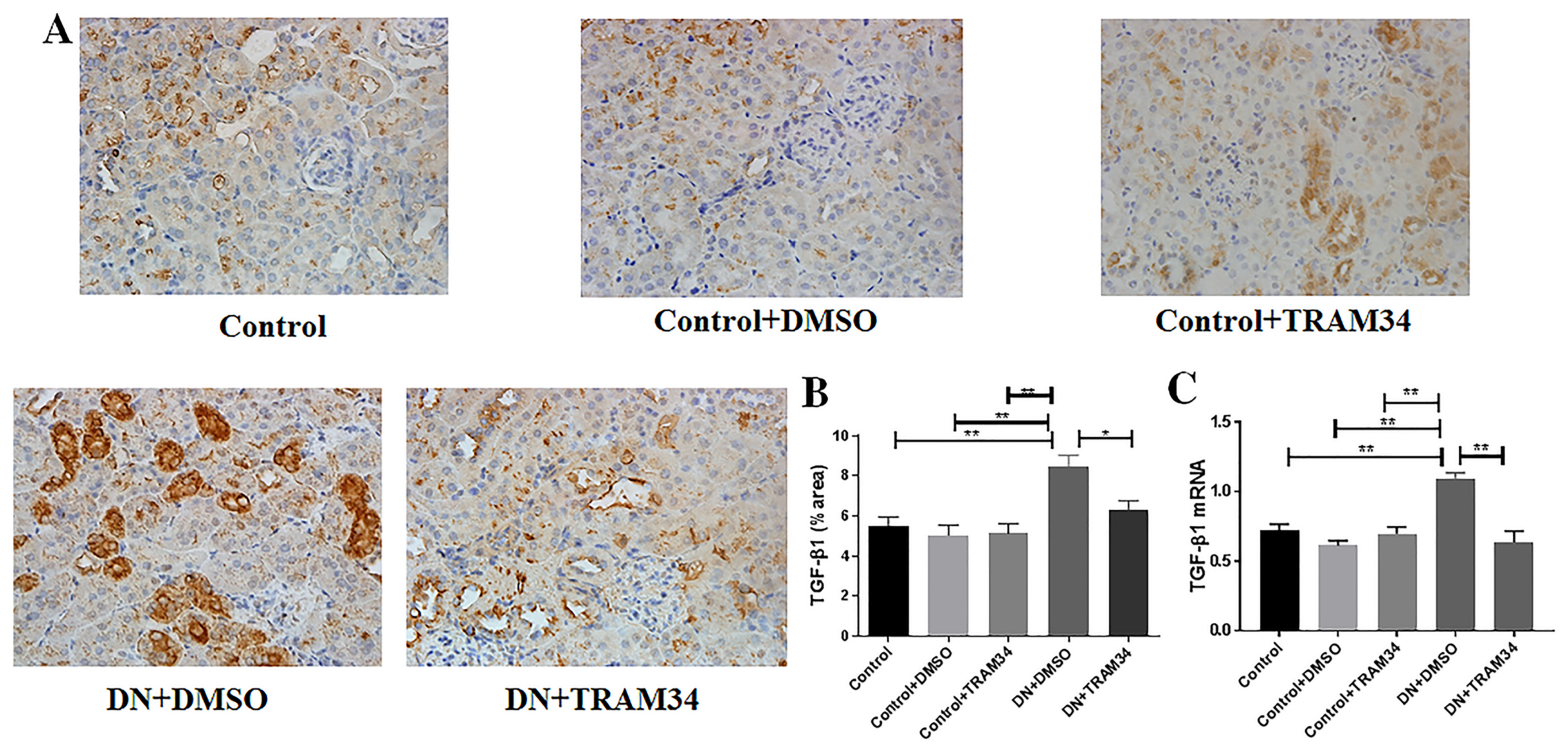
KCa3.1 blocker TRAM34 suppressed the overexpression of TGF-β1 in mice with established diabetic nephropathy. **A**. Immunohistochemical analysis showed increased TGF-β1 expression in mice with diabetic nephropathy mice compared to control mice, which was suppressed by TRAM34 **B**. The quantitation of TGF-β1 expression in mice kidneys. **C**. Quantitative RT-PCR showed increased mRNA expression of TGF-β1 mice with diabetic nephropathy compared to control mice but reduced with TRAM34 treatment. Results are presented as mean ± SEM. *P<0.05, **P<0.01. Original magnification: × 400. N = 5–8.

**Fig 8:**
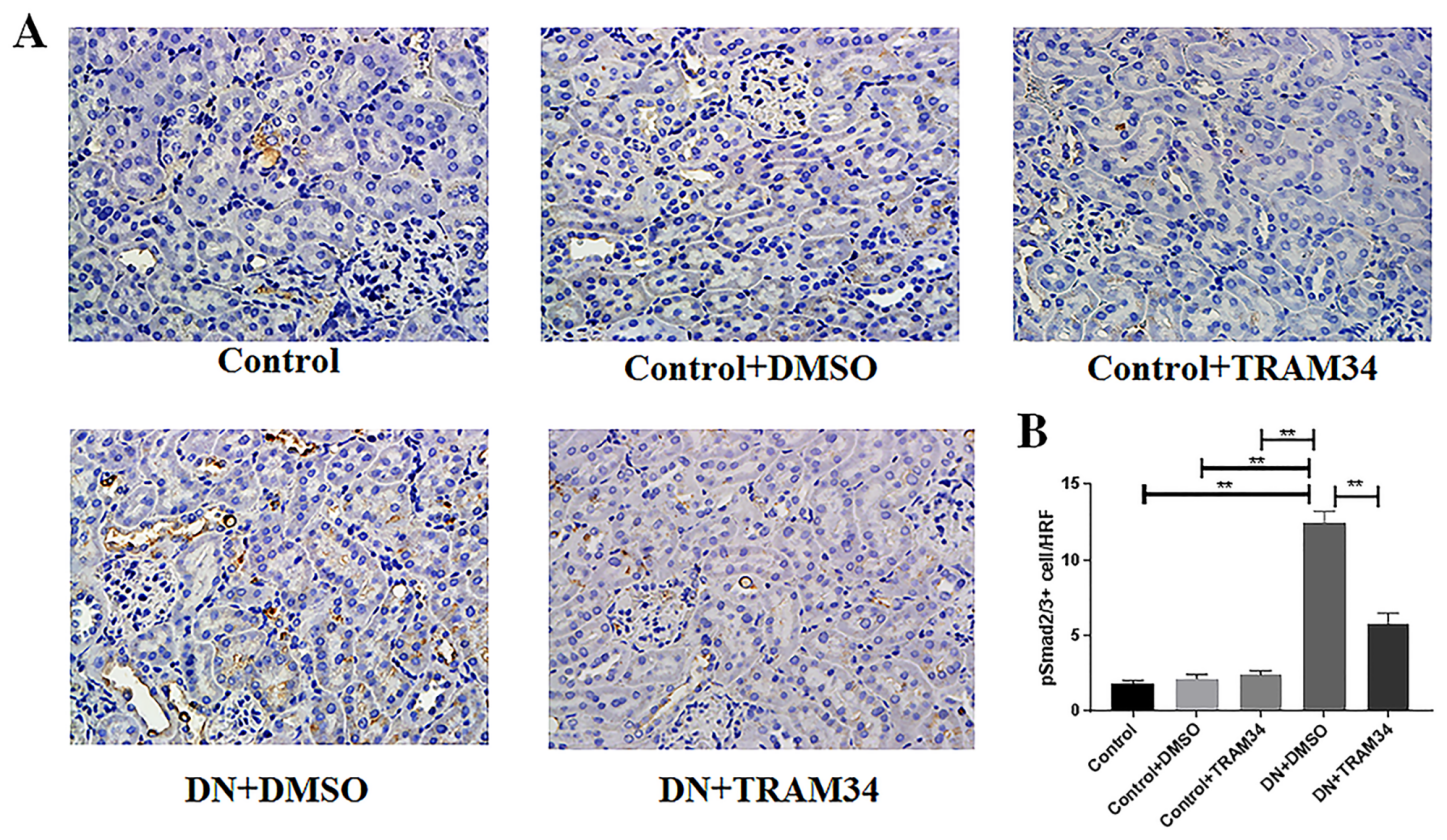
KCa3.1 blocker TRAM34 inhibited the phosphorylation of Smad2/3 in mice with established diabetic nephropathy. **A**. Immunohistochemical analysis showed increased phosphorylation of Smad2/3 in mice with diabetic nephropathy compared to control which was reversed by treatment with TRAM34. **B**. The quantitation of pSmad2/3+ cells in mice kidneys. Results are presented as mean ± SEM. **P<0.01. Original magnification: × 400. N = 5–8.

To determine whether the TGF-β1/Smad2/3 signaling pathway is an essential intermediary in KCa3.1 mediated diabetic nephropathy, we examined the effects of KCa3.1 on the expression of TGF-β1 in kidneys of mice with established diabetic nephropathy using real-time PCR and immunohistochemical staining. At both mRNA and protein levels, expression of TGF-β1 was significantly increased in mice with diabetic nephropathy (DN+DMSO) when compared with control mice (Control). Blockade of KCa3.1 with TRAM34 significantly reduced their levels in the mice with diabetic nephropathy (DN+TRAM34) (*P*<0.01, Fig 7). Phosphorylation of Smad2/3 and its subsequent nuclear translocation are critical steps in TGF-β1 signaling. Therefore, the TGF-β1-induced Smad2/3 signaling pathway was examined. As shown in Fig 8, immunohistochemical staining showed increased phosphorylation of Smad2/3 in mice with diabetic nephropathy (DN+DMSO) when compared to the control group (Control), which was suppressed by TRAM34 (*P*<0.01, Fig 8). These data indicate that the therapeutic effect of KCa3.1 in diabetic nephropathy is at least partly through inhibition of the TGF-β1/Smad2/3 signaling pathway.

### KCa3.1 blocker TRAM34 did not cause significant structural changes in the liver, heart and spleen

**Fig 9:**
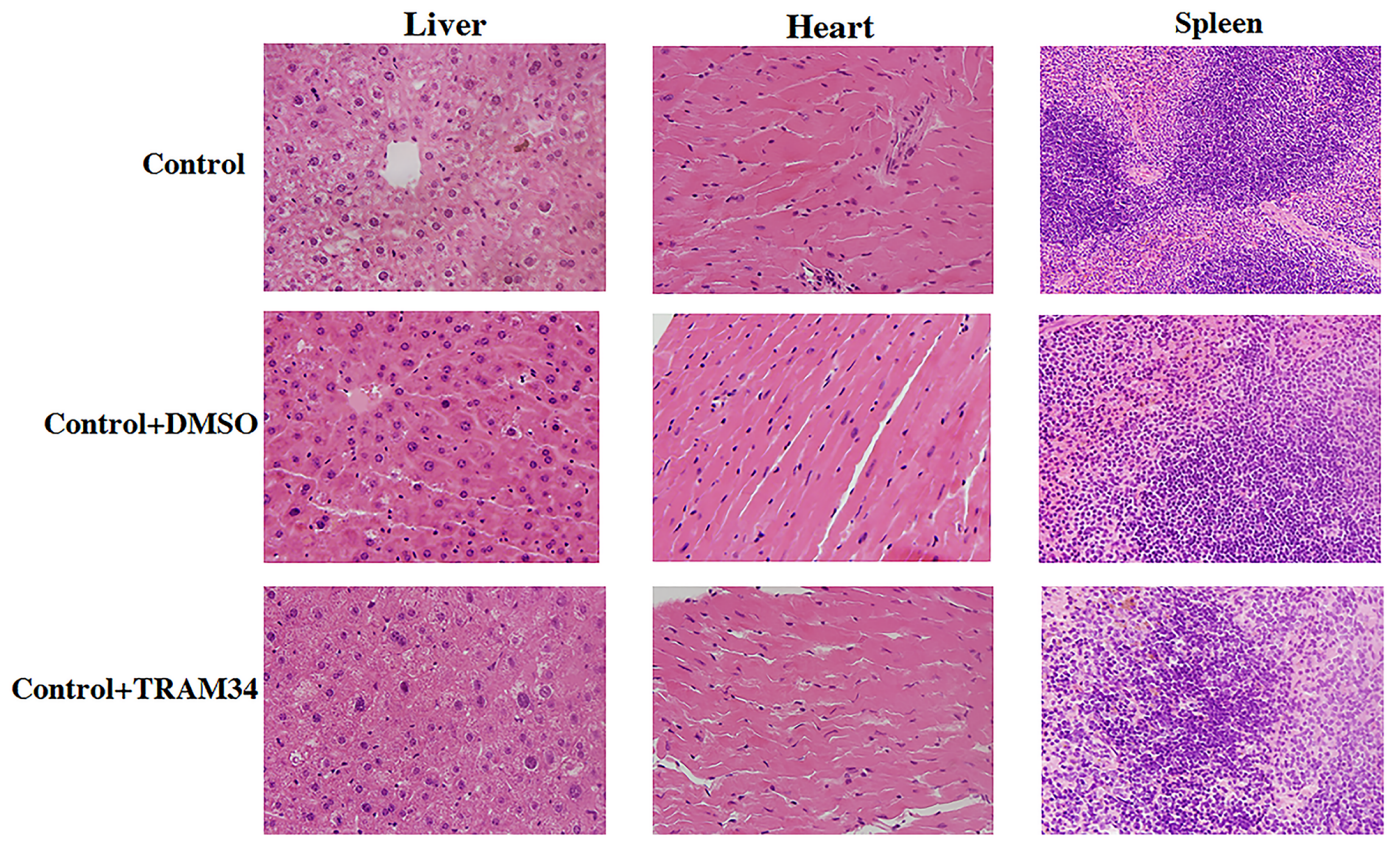
Histology of liver, heart and spleen of mice with TRAM34 treatment. Effect of TRAM34 on histological structure of liver, heart and spleen in the mice. Hematoxylin & eosin staining showed that TRAM34 did not cause significant structural changes in the liver, heart and spleen compared to normal controls. Original magnification: × 400. N = 5–8.

To determine whether TRAM34 administration for 14 weeks results in off-target toxicity in mice, liver, heart and spleen tissue samples were obtained and examined for histopathological abnormalities compared to controls. As shown in Fig 9, Hematoxylin & eosin staining showed that TRAM34 did not cause significant structural changes in the liver, heart and spleen compared to normal controls. These results are reassuring with respect to the safety profile of KCa3.1 blockade.

## Discussion

In clinical practice, patients with diabetes mellitus develop chronic kidney disease manifesting as albuminuria and decreased estimated Glomerular Filtration Rate (eGFR), alone or in combination. The pathology of advanced diabetic nephropathy is characterized by glomerular and tubulointerstitial lesions, including diffuse and nodular mesangial expansion and glomerular basement membrane thickening, tubular atrophy, interstitial inflammation, and extracellular matrix deposition, which are closely related to renal functional loss and progression towards ESKD [25]. To date, ‘best practice’ therapy includes optimal blood pressure and glycemic control, and treatment directed to a reduction in albuminuria [2]. However, such therapies have only slowed the progression to ESKD, and strategies to reverse established diabetic nephropathy are not available. Therefore, effective strategies to reverse structural and functional abnormalities are required, Targeting strategies to prevent renal fibrosis are particularly attractive as this is the key pathological feature portending renal functional decline. Hence therapeutic strategies to reverse and not only prevent renal damage should be identified. The present study has demonstrated that blockade of KCa3.1 with the pharmacological inhibitor TRAM34 at least partially improved albuminuria and reversed the pathological changes evident in rodent diabetic nephropathy.

KCa3.1 blockade and deficiency has been shown to prevent the onset and development of renal injury in animal models of obstructive [19] and diabetic nephropathy [11, 20, 22, 23]. To examine whether KCa3.1 blockade is able to reverse established diabetic nephropathy, an eNOS-/- mouse model of diabetic nephropathy was established as previously described [11] and then treated with the selective pharmacological inhibitor of KCa3.1, TRAM34 [26]. As reported, animals constitutively lacking KCa3.1 are of normal appearance, reproduce normally and have no abnormalities of any major organs. Hence pharmacological inhibition of KCa3.1 with TRAM34 in mice is not considered to lead to significant adverse effects [9]. Our previous studies demonstrated that diabetic eNOS-/- mice, induced by STZ, developed significant impairment of renal function and clear renal pathological changes at 24 weeks of diabetes [11]. In this study, diabetic nephropathy was established in eNOS-/- mice after diabetes induction by STZ for 24 weeks as per previous studies [11] and then injected with TRAM34 subcutaneously for 14 weeks. The results demonstrated that TRAM34 reversed renal injury (Table 1) and normalized KCa3.1 expression compared to vehicle-treated diabetic nephropathy mice (Fig 1).

It is well recognized that chronic hyperglycemia-induced sterile inflammation has a prominent role in initiation of the fibrotic process [27]. CD68+ macrophages contribute to renal inflammation, which initiates renal fibrosis [28]. Our results have shown TRAM34 significantly suppressed icreased CD68+ cells in the mice with diabetic nephropathy (Fig 2). F4/80+ macrophages also play an important role in various forms of renal injury including diabetic nephropathy as demonstrated previously by our group [11]. In this study, our results have demonstrated that TRAM34 significantly suppressed icreased F4/80+ cells in the mice with diabetic nephropathy (Fig 3). Collectively, both CD68+ and F4/80+ macrophages, which are known to contribute to renal inflammation, were significantly suppressed in diabetic nephropathy kidneys by TRAM34 administration.

It is well accepted that extracellular matrix deposition is the most striking feature in diabetic renal glomerular and interstitial fibrosis [7, 8]. The increased matrix protein production and decreased protein degradation leads to deposition of extracellular matrix proteins, including collagens and fibronectin in the mesangium and tubular interstitium [29]. Our previous studies have uniformly shown excessive extracellular matrix deposition in diabetic kidneys [11, 20]. Our results in this study have shown TRAM34 normalized expression of type I collage and fibronectin in diabetic nephropathy kidneys (Figs 5 and 6), which are key extracelluar matrix proteins contributing to renal fibrosis.

It is well known that hyperglycaemia triggers cellular responses that promote tubulointerstitial fibrosis, with TGF-β1 being a central mediator of pathology. High glucose-induced TGF-β1 [30–34] and downstream signaling via the Smad pathways play essential roles in the development of renal fibrosis in diabetic and other forms of nephropathy [35]. Targeting TGF-β/Smad signalling may represent a specific and effective therapy for chronic kidney disease associated with renal fibrosis [36]. TGF-β1 induced renal fibrosis has been well reported [36]. Conversely, inhibition of TGF-β1 by multiple strategies suppresses renal fibrosis and prevents progressive loss of kidney function [37]. However direct targeting of TGF-β1 is unlikely to yield a viable antifibrotic therapy due to the involvement of TGF-β1 in other processes [37]. For example, regulatory T cells utilize TGF-β to paralyze cell activation and differentiation to suppress immune response. Hence inhibition of TGF-β in regulatory T cells could aggravate autoimmune disease [38]. TGF-β1 signaling is essential for wound healing, including both non-specific scar formation and tissue-specific regeneration. We have shown in this study TRAM34 normalized the expression of TGF-β1 and phosphorylation of Smad2/3 in the mice kidneys with diabetic nephropathy (Figs 7 and 8), which indicates TRAM34 repaired diabetic kidney damage partially through inhibiting the TGF-β1 signaling pathway. To date, the mechanism of tissue repair is unclear. In general, the balance of tissue inhibitors of metalloproteinases (TIMPs) and matrix metalloproteinases (MMPs) regulates extracellular matrix degradation and remodeling in development and disease [39]. However, both TIMPs and MMPs exert differential roles at the early and late stage of kidney fibrosis, which leads the complex of tissue repair process in kidney fibrosis. It was recently reported that the tumour necrosis factor superfamily member glycoprotein OX40 ligand (OX40L) exerts potent profibrotic effects by promoting the infiltration of inflammatory cells into lesional tissues and therefore the release of proinflammatory mediators, thereafter leading to fibroblast activation, and OX40L blockade induced the regression of established fibrosis in mouse models of the disease [40]. The OX40L pair is involved in late T-cell costimulatory signaling. It is reported that Kv1.3 and KCa3.1 cooperatively and compensatorily regulate antigen-specific memory T cell function and inhibition of KCa3.1 ameliorate T cell-mediated intestinal inflammatory disease such as colitis [41]. We had previously shown KCa3.1 blockade suppressed inflammatory response by reversing MCP-1, ICAM1, and F4/80 expression in diabetic kidneys [11]. KCa3.1 blockade may contribute to the renal damage repair through blocking OX40L. As recently summarised in a review article that the repair response triggered by core immune pathways is context-dependent and is a function of a combination of multiple determinants, including the nature of the damage, the amplitude and duration of different polarization states of inflammatory cells, and the intrinsic regenerative capacity of different organs and tissues [42]. Finally, 14-weeks administration of TRAM34 did not cause significant pathological changes in other organs including heart, liver and spleen (Fig 9).

In summary, we have uniquely demonstrated that blockade of KCa3.1, using the pharmacological inhibitor TRAM34, improves clinicopathological evidence of diabetic nephropathy. In particular it reduced albuminuria, limited sterile inflammation and reversed extracellular matrix deposition in mice kidneys with diabetic nephropathy. Mechanistically it inhibited the TGF-β1 signaling pathway. However the pathways that mediate cellular repair are yet to be elucidated. It is still unknown why scars are capable of resolving in some tissues such as liver, but not in others. Understanding the differences in macrophage origin, recruitment, and activation in injured tissue and the interation of these cells with extracellular matrix deposition in the wound environment may explain the variation of scar repair between different organs and species [42]. These studies importantly demonstrate that renal functional decline and pathological changes of established diabetic nephropathy can be ameliorated with blockade of the KCa3.1 channel.
